# Association of neutrophil-lymphocyte ratio and T lymphocytes with the pathogenesis and progression of HBV-associated primary liver cancer

**DOI:** 10.1371/journal.pone.0170605

**Published:** 2017-02-23

**Authors:** Xiaoli Liu, Lingling He, Junyan Han, Lijia Wang, Mengge Li, Yuyong Jiang, Xianbo Wang, Zhiyun Yang

**Affiliations:** 1 Center for Integrative Medicine, Beijing Ditan Hospital, Capital Medical University, Beijing, P.R. China; 2 Institute of Infectious Diseases, Beijing Key Laboratory of Emerging Infectious Diseases, Beijing Ditan Hospital, Capital Medical University, Beijing, P.R. China; Universita degli Studi di Pavia, ITALY

## Abstract

**Background:**

The neutrophil–lymphocyte ratio (NLR) is a new prognostic predictor for patients with liver cancer. The association of NLR and T lymphocytes with the pathogenesis and progression of liver cancer is poorly understood.

**Methods:**

Seventy-three patients with hepatitis B virus (HBV)-associated primary liver cancer (HBV-PLC), 50 patients with HBV-associated liver cirrhosis (HBV-LC) and 37 patients with chronic HBV infection (CHB) were prospectively enrolled from July 1, 2013 to February 28, 2014 in Beijing Ditan Hospital, Capital Medical University (Beijing, China). The NLR, proportions and concentrations of neutrophils and lymphocytes, concentration of subpopulations of lymphocytes, and the expression of CD31 (index for recent thymic output) and HLA-DR (index for activation of T lymphocytes) of T cells in the peripheral blood samples of the patients were assessed and statistically compared between different groups.

**Results:**

The NLR was significantly increased from patients with CHB, those with HBV-LC to those with HBV-PLC (P<0.05), along with significant increase of neutrophils and decrease of lymphocytes in the same way (P<0.05). The concentrations of T lymphocytes, natural killer cells, B cells, CD4^+^ T cells and CD8^+^ T cells were decreased from patients with CHB, those with HBV-LC to those with HBV-PLC, and were significantly reduced in patients with HBV-PLC compared with those in patients with CHB (P<0.05). The CD31 and HLA-DR expression of naive CD4^+^ and CD8^+^ T cells was significantly decreased and increased, respectively in patients with HBV-PLC compared with that in patients with CHB.

**Conclusions:**

Elevated NLR, resulted from the increase of neutrophils and decrease of lymphocytes, is positively associated with the pathogenesis and progression of HBV-PLC. The reduced thymic output and hyperactivation of T lymphocytes may contribute to the decrease of T lymphocytes, which could be also related to the pathogenesis of HBV-PLC.

## Introduction

Primary liver cancer (PLC) mainly consisting of hepatocellular carcinoma (HCC) is the fifth most common cancer worldwide [1] and the second most common cause of cancer mortality in men and the sixth in women [2]. Chronic hepatitis B virus (HBV) infection is a major risk factor for liver cirrhosis (LC) and HCC, which leads to over 80% of the total cases of PLC in China where more than 7% of the whole population is infected with HBV [3,4].

HBV-associated PLC (HBV-PLC) is considered to be an inflammatory cancer. Neutrophil to lymphocyte ratio (NLR) is a good laboratory marker to evaluate systemic inflammation. Preoperative and postoperative NLR had been used as a poor prognostic factor for the treatment of various types of cancer such as liver cancer [5–10]. A recent study showed that the significant association of preoperative NLR with poor prognosis of HCC patients was only applied to TNM stage I instead of stage II or III HCC [9]. Until now, however, the dynamic changes of NLR during the pathogenesis and progression of liver cancer have not yet been fully elucidated.

Thymic output provides the naive T cells essential for the normal functioning of T cell-dependent immunity such as anti-cancer protection. Previous reports showed impaired recent thymic output function in patients with cancers such as lymphoma, head and neck cancer and glioblastoma multiforme cancer [11–15]. However, the association of recent thymic output with the pathogenesis of HBV-PLC is still unclear.

In this study, we enrolled patients with chronic HBV infection (CHB), those with LC and those with HBV-PLC to observe the dynamic changes of NLR in the pathogenesis and progression of HBV-PLC. We then further observed the changes of neutrophils, lymphocytes and the subpopulations, recent thymic output and the status of activation of T lymphocytes during the pathogenesis of HBV-PLC.

## Materials and methods

### Patients

Seventy-three patients with HBV-PLC (HBV-PLC group), 50 patients with LC due to HBV (HBV-LC group) and 37 patients with CHB (CHB group) were prospectively enrolled from July 1, 2013 to February 28, 2014 in Beijing Ditan Hospital, Capital Medical University (Beijing, China). This study was approved by the Ethnics Committee of Beijing Ditan Hospital, Capital Medical University. Written informed consent was obtained from each patient. Information that could identify individual participants during or after data collection could not be accessed. Inclusion criteria of patients were: 1) aged 18–75 years, 2) positive for serum hepatitis B surface antigen (HBsAg) and/or positive for HBV DNA, 3) without any antiviral treatment within 6 months prior to the experiment. Exclusion criteria included: 1) patients combined with hepatitis C virus and/or human immunodeficiency virus infection or combined with alcoholic liver disease, autoimmune hepatitis, inherited metabolic liver disorders, drug-induced liver diseases, severe fatty liver or other chronic liver diseases, 2) patients with metastatic liver cancer or other types of cancer, 3) pregnant or lactating women. HBV-PLC was diagnosed based on the histologic examination of tumor tissues, or serum concentration of α-fetoprotein (AFP) ≥ 400 ng/mL in combination with radiologic evidence of space-occupying lesion(s) in the liver.

### Assessment of peripheral blood T lymphocyte subsets

Five ml peripheral blood samples from patients within 2 days after the admission to hospital and before the treatments such as radiofrequency ablation (RFA), transcatheter arterial chemoembolization (TACE) and hepatectomy were collected into the EDTA anticoagulant-coated tubes. One hundred and thirty μl whole blood was sucked, deleted the red blood cells using FACS^TM^ Lysing Solution (BD Biosciences, San Jose, CA, USA), stained with CD3, CD4, CD8, CD31, CD45RA and HLA-DR antibodies (all antibodies were purchased from BD Biosciences), respectively, and then underwent flow cytometric analysis by using a four-color FACSCalibur flow cytometer (BD Biosciences). For the determination of T, natural killer (NK) and B lymphocyte cells, 50 μl whole blood was sucked and stained with 20 μl MultiTEST CD45-Percp/CD3-FITC/CD4-APC/CD8-PE TruCount four-color kit (for T cells) or MultiTEST CD45-Percp/CD3-FITC/CD16+CD56-PE/CD19-APC TruCount four-color kit (for B and NK cells) (BD Biosciences). Isotype-matched antibodies were used as negative controls. Data were analyzed by Flowjo software (Treestar, Ashland, OR, USA). Neutrophils and lymphocytes were determined by the blood routine examination, and were expressed as the percentages among the total white blood cells and also as cells number per L peripheral blood. CD3^-^CD16^+^CD56^+^ NK cells, CD3^-^CD19^+^ B cells, CD3^+^CD4^+^ T cells and CD3^+^CD8^+^ T cells were determined as cell number per μL peripheral blood.

### Statistical analysis

The normality of data was evaluated using the Kolmogorov-Smirnov test. Normally distributed data were expressed as mean± standard error of the mean (SEM), and abnormally distributed data were expressed as median ± interquartile range (IQR). Data were analyzed using GraphPad5 (GraphPad Software, La Jolla, CA, USA) or SPSS for Windows V.13.0 (SPSS Inc. Chicago, IL, USA). Normally distributed data were compared between the CHB, HBV-LC and HBV-PLC groups by using one-way ANOVA followed by Bonferoni test. p<0.05 was considered statistically significant. For data not normally distributed, comparison between the three groups was made using one-way Kruskal–Wallis test (p<0.05 was considered statistically significant) followed by a post-hoc Mann–Whitney test, with corrections of p values according to Bonferroni (p<0.017 was considered statistically significant).

## Results

### Patient basic characteristics

Patient basic demographic, clinical and laboratory characteristics were summarized in Table 1. There was no significant difference in gender among the CHB, HBV-LC and HBV-PLC groups. The mean ages of HBV-LC (51.2±8.5 years) and HBV-PLC (54.6±10.1 years) groups were similar (P = 0.062), which were both significantly older than that of CHB group (36.0±14.4 years, both p<0.001). The serum levels of alanine aminotransferase, aspartate aminotransferase, prothrombin activity, albumin, and HBV DNA in patients with HBV-LC and those with HBV-PLC patients were significantly lower than in CHB patients (all P<0.05). The model for end-stage liver disease score in HBV-LC or HBV-PLC patients was significantly higher than in CHB patients (P<0.05). For Child-Pugh grade, patients with HBV-LC and those with HBV-PLC had significantly higher percentages of more severe grades (i.e. B and C) than patients with CHB (p<0.001). Since Okuda staging system was only for HBV-PLC and there was no such data for the other two groups, no similar comparison between the three groups was shown.

**Table 1:** Patient basic characteristics.

Characteristics	CHB (n = 37)	LC (n = 50)	PLC (n = 73)	P value
**Age (year)**	36.0±14.4	51.2±8.5***	54.6±10.1***	<0.001
**Male %**	64.86%	66%	76.71%	0.28
**Serum alanine aminotransferase, (U/L)**	172.5 (43.6–342.0)	30.7 (20.9–57.8)*	29.7 (16.9–51.2)*	<0.001
**Serum aspartate aminotransferase (U/L)**	92.2 (40.7–145.0)	39.9 (24.6–70.7)*	40.2 (23.4–87.8)*	0.007
**Serum total bilirubin (μmol/L)**	18.0 (10.5–28.3)	25.8 (14.3–38.1)	20.8 (13.9–40.4)	0.121
**Serum albumin (g/L)**	39.2±4.6	32.9±6.8*	34.8±6.3*	<0.001
**HBV DNA (log copies/mL)**	4.64±1.64	2.52±1.83***	2.77±1.18***	<0.001
**Alpha fetal protein (ng/mL)**	9.5 (3.6–88.8)	4.7 (2.3–14.9)*	34.4 (4.2–735.5)^§§§^	<0.001
**Serum prothrombin activity (%)**	95.4±21.3	67.9±20.7*	76.1±18.1*^§§§^	0.001
**Model for end-stage liver disease score**	4.0±4.1	7.8±6.1*	8.1±7.9*	0.01
**Child-Pugh grade**				<0.001
**A (n/%)**	83.8% (31/37)	24% (12/50)	43.8% (32/73)	
**B (n/%)**	16.2% (6/37)	64% (32/50)	39.7% (29/73)	
**C (n/%)**	0	12% (6/50)	16.4% (12/73)	
**Okuda stage**				
**I (n/%)**			21	
**II (n/%)**			36	
**III (n/%)**			16	

HBV: Hepatitis B Virus.

*p<0.05

***p<0.001 compared with CHB group

^§§§^p<0.001 compared with HBV-LV group.

### Elevated NLR in patients with HBV-PLC

**Fig 1:**
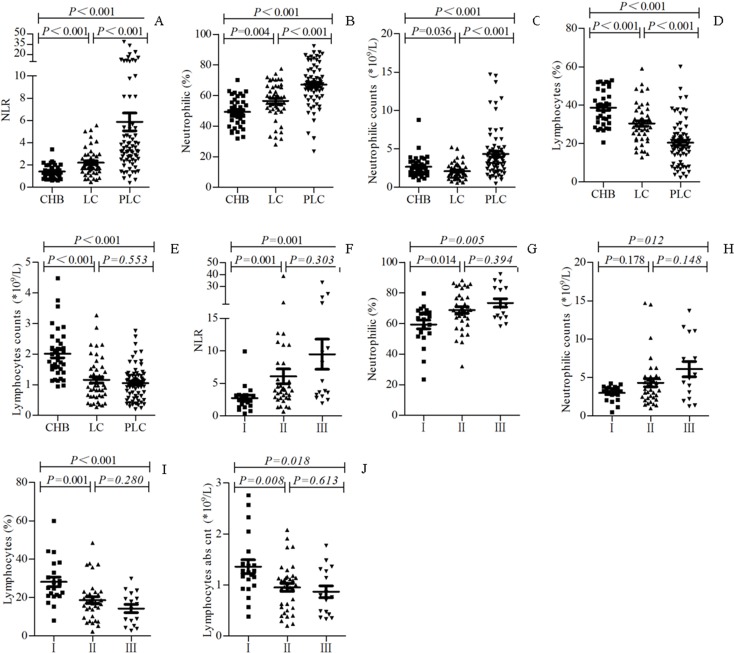
Elevated NLR in patients with HBV-PLC. NLR (A), percentage (B) and concentration (C) of peripheral blood neutrophils, and percentage (D) and concentration (E) of peripheral blood lymphocytes were compared between patients with CHB, those with HBV-LC and those with HBV-PLC. In addition, NLR (F), percentage (G) and concentration (H) of peripheral neutrophils, and percentage (I) and concentration (J) of peripheral lymphocytes were compared between patients with Okuda I, II and III HBV-LC. p<0.05 was considered statistically significant. abs cnt: absolute count.

To see the dynamic changes of NLR in the pathogenesis and progression of PLC, we analyzed the NLR from patients with CHB, HBV-LC and HBV-PLC, respectively. The NLR and percentage and concentration of neutrophils were significantly increased from CHB, HBV-LC to HBV-PLC groups in turn (all P<0.05, Fig 1A–1C), while the percentage and concentration of lymphocytes were decreased in turn from CHB, HBV-LC to HBV-PLC groups (most of P<0.001, Fig 1D and 1E). In addition, the NLR and percentage and concentration of neutrophils were increased from Okuda I, II to III HBV-PLC in turn, and were significantly higher in Okuda III than in I and II HBV-PLC (P<0.05, Fig 1F–1H). In contrast, percentage and concentration of lymphocytes were decreased from Okuda I, II to III HBV-PLC, and were significantly lower in Okuda III than in I and II HBV-PLC (P<0.05, Fig 1I and 1J).

### Decreased peripheral lymphocyte subpopulations in patients with HBV-PLC

**Fig 2:**
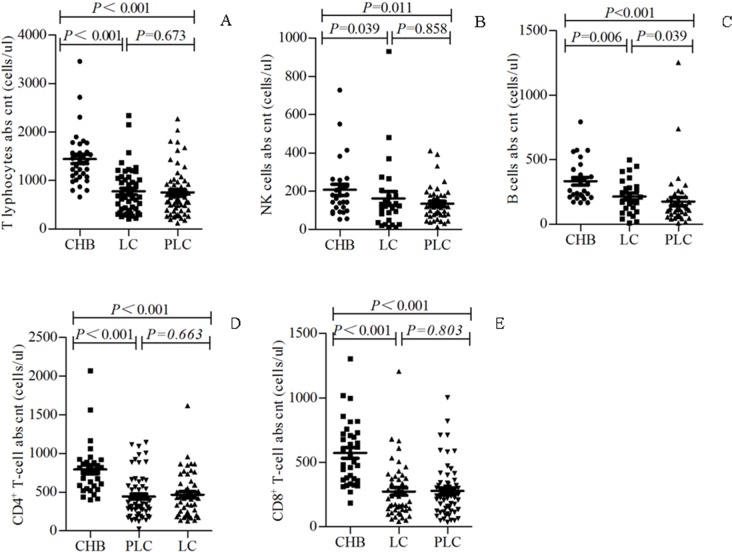
Decreased peripheral lymphocyte subpopulations in patients with HBV-PLC. The concentrations of the peripheral blood T lymphocytes (A), NK cells (B), B cells (C), CD4^+^ T cells (D), CD8^+^ T cells (E) were compared between patients with CHB, those with HBV-LC and those with HBV-PLC. p<0.05 was considered statistically significant. abs cnt: absolute count.

We further analyzed the changes of the detailed lymphocyte subpopulations in patients with HBV-PLC. As shown in Fig 2, the concentrations of CD3^+^ T lymphocytes, NK cells, B cells, CD4^+^ T cells and CD8^+^ T cells were decreased from patients with CHB, those with HBV-LC to those with HBV-PLC, and were significantly decreased in patients with HBV-LC and those with HBV-PLC compared with those with CHB (all P<0.05). However, no significant difference was observed between patients with HBV-PLC and those with HBV-LC (P>0.05).

### Decreased peripheral recent thymic output in patients with HBV-PLC

**Fig 3:**
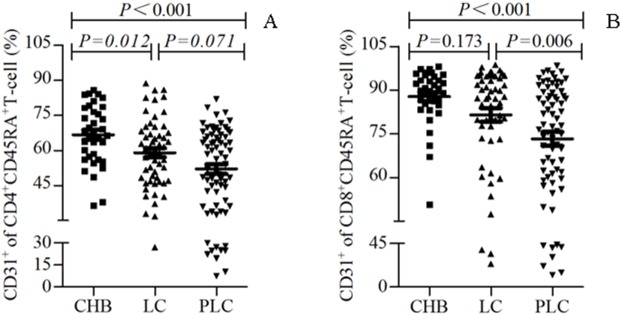
Decreased peripheral recent thymic output in patients with HBV-PLC. Percentages of CD31^+^ -expressed cells (CD31%) among the CD45RA^+^CD4^+^ T cells (A) and CD45RA^+^CD8^+^ T cells (B) of the peripheral blood were compared between the patients with CHB, those with HBV-LC and those with HBV-PLC. p<0.05 was considered statistically significant.

Since CD31 expression is highly correlated with T-cell receptor rearrangement excision circles [16], a useful marker for recent thymic output, we then observed the recent thymic immigrant T cells of patients through the assessment of the percentage of CD31^+^ -expressed cells (CD31%, expression of CD31) among the CD45RA^+^CD4^+^ T cells and CD45RA^+^CD8^+^ T cells (i.e. CD31^+^CD45RA^+^CD4^+^ T cells/CD45RA^+^CD4^+^ T cells %, and CD31^+^CD45RA^+^CD8^+^ T cells /CD45RA^+^CD8^+^ T cells %, respectively). It was found that CD31 expression of CD4^+^ T cells and that of CD8^+^ T cells were decreased from patients with CHB, those with HBV-LC to those with HBV-PLC. CD31 expression of CD4^+^ T cells was significantly decreased in patients with HBV-LC and those with HBV-PLC compared with CHB patients (both P<0.05, Fig 3), while no significant difference was observed between the patients with HBV-LC and those with HBV-PLC (P>0.05). The CD31 expression of CD8^+^ T cells in patients with HBV-PLC was significantly lower than in patients with CHB and those with HBV-LC (P<0.05, Fig 3). This result showed impaired recent thymic output in patients with HBV-PLC. Furthermore, the decreased thymus production was consistent with the less naive CD4^+^ and CD8^+^ T cells in the HBV-PLC patients (data not shown).

### Hyperactivation of peripheral CD4^+^ and CD8^+^ T cells in patients with HBV-PLC

**Fig 4:**
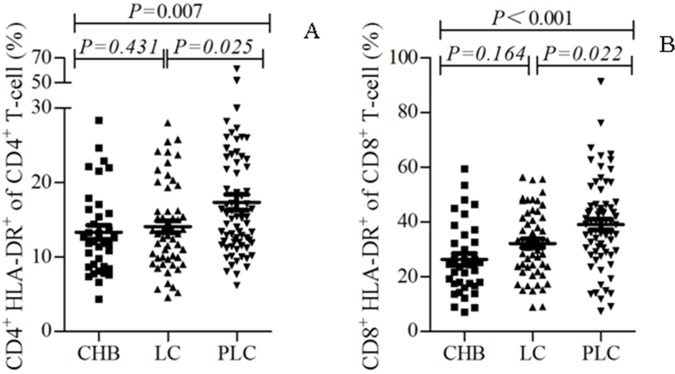
Hyperactivation of peripheral CD4^+^ and CD8^+^ T cells in patients with HBV-PLC. Percentages of HLA-DR -expressed cells (HLA-DR %) among the CD4^**+**^ T cells (A) and CD8^**+**^ T cells (B) of the peripheral blood were compared between the patients with CHB, those with HBV-LC and those with HBV-PLC. p<0.05 was considered statistically significant.

The status of T-cell activation of patients was assessed through the evaluation of the expression of HLA-DR, a marker for T-cell activation, in CD4^+^ and CD8^+^ T cells. The percentage of CD4^+^HLA-DR^+^ cells (referred to expression of HLA-DR) among CD4^+^ cells and that of CD8^+^ HLA-DR^+^ cells (also referred to expression of HLA-DR) among CD8^+^ T cells were elevated from patients with CHB, those with HBV-LC, to those with HBV-PLC (Fig 4). The expressions of HLA-DR among CD4^+^ T cells and CD8^+^ T cells were significantly increased in patients with HBV-PLC compared with those with CHB and HBV-LC, respectively (P<0.05, Fig 4). This result indicates that the expression of HLA-DR was highly increased and thus further showed that CD4^+^ and CD8^+^ T cells were highly activated in patients with HBV-PLC.

## Discussion

NLR is considered to reflect the systemic inflammatory response in patients with cancers [17, 18]. Previous reports showed that preoperative NLR is of strong prognostic significance for patients with HCC as an inflammatory cancer [4, 19, 20]. However, the association of NLR with the pathogenesis and progression of HBV-PLC is unclear. In East Asia, especially in China, liver cancer is mainly caused by HBV infection and cirrhosis, which is developed from chronic HBV infection, LC to PLC [21, 22]. Therefore, in this study we enrolled patients with CHB, HBV-LC and HBV-PLC, respectively to simulate the pathogenesis process of HBV-PLC. We showed NLR was significantly increased in an order from CHB, HBV-LC to HBV-PLC patients, which was attributable to the significant increase of neutrophils from CHB, HBV-LC to HBV-PLC patients in turn but decrease of lymphocytes successively. This study, from the clinical perspective, indicates that elevation of NLR, resulted from the increase of peripheral neutrophils and reduction of lymphocytes, is associated with the pathogenesis of HBV-PLC. We also showed that the NLR was increased from Okuda I, II to III HBV-PLC in turn and particularly was significantly higher in Okuda III than in I and II HBV-PLC, indicating NLR is positively associated with the progression of HBV-PLC. Further we showed impaired recent thymic output and hyperactivation of T lymphocytes in HBV-PLC patients.

The elevation of NLR indicated more severe systemic inflammatory response in HBV-PLC patients. Lymphocytes are vital important for immune response. T lymphocytes comprise CD4^+^ helper T (Th) cells, CD8^+^ cytotoxic T (CTL) cells, etc. Th cells play a role in increasing and boosting antitumor immune response through promoting the production of antibodies in B cells and inducing the differentiation of CD8 T cell into CTL cells [23,24]. CTL cells recognize tumor antigens and directly clear tumor cells, thus displaying significant antitumor effects [25]. Abnormal concentrations of T lymphocyte subpopulations were observed in the non-small cell lung cancer, head and neck cancer, and ovarian cancer [26–28]. Similarly, we further showed the concentrations of T lymphocytes, B cell, NK cells, CD4^+^ T cells and CD8^+^ T cells were decreased from patients with CHB, those with HBV-LC to those with HBV-PLC, and were significantly reduced in patients with HBV-PLC compared with those in patients with CHB. This result reflects the disturbance and suppression of T cells, which may be associated with a poor immune response in HBV-PLC patients. NK cells secrete cytokines (e.g. IFN-γ) and chemokines that influence the host’s immune response, and/or kill virus-infected cells via perforin/granzyme or death receptor–related pathways (e.g. Fas, tumor necrosis factor-related apoptosis-inducing ligand) [29,30]. B and T lymphocytes are mainly involved in the antibody production and cell-mediated immune responses, respectively. The decreased concentrations of lymphocytes (including NK cells, B and T lymphocytes) of patients with HBV-PLC in our study reflected the impaired anti-tumor and anti-virus responses in HBV-PLC patients in comparison with that in those with CHB and HBV-LC, respectively.

The correlation of NLR with tumor stages is still controversial. In some studies, it was shown that NLR was significantly increased with more advanced tumor stages in neuroendocrine tumors [31], lung adenocarcinoma [32], laryngeal squamous cell carcinoma [33], endometrial cancer [34], gastric cancer [35], small cell lung cancer [36], etc. Particularly, the preoperative NLR increased with the advanced Barcelona clinic liver cancer stages and tumor-node-metastasis (TNM) stages [37]. However, Duzlu et al. revealed a non-significant elevation of NLR with increasing tumor stage in larynx carcinoma [38]. A recent meta-analysis showed that increased NLR was not significantly correlated with pathological stage of prostate cancer [39].

Okuda staging system collectively cares the tumor conditions and liver functions of patients, while in contrast, Union for International Cancer Control (UICC) TNM system reflects the tumor biological characteristics and associated factors with the prognosis of liver cancer after the surgical resection, without concerning the liver function, which thus may not be so suitable for the assessment of the conditions of patients with relatively advanced liver cancer. In this study, some patients were unsuitable for the surgical resection and thus received TACE, RFA, etc., which could not be adequately assessed by UICC TNM staging system. Since we cared more about the general conditions of patients, we used Okuda instead of UICC TNM staging system in this study. We demonstrated that the NLR was increased from Okuda I, II to III HBV-PLC, suggesting NLR is positively correlated with the progression of HBV-PLC. Our study was generally consistent with some results reported [31–37]. This might be related to the result that patients with more advanced stages had higher concentration of peripheral neutrophils but lower concentration of peripheral lymphocytes.

The human naive CD31^+^ T cells contain cell populations recently emigrated from the thymus. A previous study showed that the analysis of CD31-expressed naive CD4^+^ T cells could be used as a marker to identify potential low responders or non-responders with respect to primary immune responses against pathogens [40]. We showed that the recent thymus output function, reflected by the expression of CD31 of CD4^+^ T cells and that of CD8^+^ T cells, was decreased from patients with CHB, those with HBV-LC to those with HBV-PLC, and was significantly decreased in patients with HBV-PLC compared with those with CHB and those with HBV-LC. This indicates the impairment of the recent thymus output function during the pathogenesis of HBV-PLC. Furthermore, consistence of the decreased thymus production with the less naive CD4^+^ and CD8^+^ T cells in the HBV-PLC patients (data not shown) suggests that the reduction of T lymphocytes in patients with HBV-PLC may be at least partially attributable to the depressed early thymus production.

The HLA-DR antigen which appears on T cells after activation is used as a marker for T-cell activation. We showed the status of T lymphocyte activation, reflected by the expression of HLA-DR of CD4^+^ T cells and CD8^+^ T cells, was elevated from patients with CHB, those with HBV-LC to those with HBV-PLC, and was significantly increased in patients with HBV-PLC compared with those with CHB and HBV-LC, respectively. This result indicates that CD4^+^ and CD8^+^ T lymphocytes cells were highly activated in patients with HBV-PLC. Hyperactivation may lead to death of T lymphocytes, which exhausts T lymphocytes and thus decreases the concentrations of CD4^+^ and CD8^+^ T lymphocytes in patients with HBV-PLC. We therefore supposed that in addition to the decreased recent thymus output, the reduction of T cells in patients with HBV-PLC might be also partially due to T lymphocyte hyperactivation.

An inevitable limitation of this study is a significant difference in age between patients in the three groups. This might affect the expression of CD31 of patients in this study. However, given the progression from CHB, HBV-LV to HBV-PLC, it is understandable that the mean age of patients with HBV-PLC is older than those with CHB and those with HBV-LC. In addition, patient number of each group was relatively small. Further study with larger sample size and variable analysis is needed to validate these results.

## Conclusion

In summary, our data suggests that the elevated NLR, resulted from the increase of neutrophils and decrease of lymphocytes, is positively associated with the pathogenesis and progression of HBV-PLC. The reduction of the recent thymic output and hyperactivation of T lymphocytes may contribute to the decrease of T lymphocytes, which could be also related to the pathogenesis of HBV-PLC. This study revealed the association of NLR, lymphocytes, recent thymic output and T lymphocyte hyperactivation with the pathogenesis of HBV-PLC from the clinical perspective, which will be beneficial for the dissociation of the mechanisms underlying the pathogenesis and progression of HBV-PLC and for the early diagnosis of HBV-PLC and the corresponding treatment as well.
